# Inaccuracies in Clinical Chemical Analysis

**DOI:** 10.6028/jres.093.067

**Published:** 1988-06-01

**Authors:** Merle A. Evenson

**Affiliations:** University of Wisconsin, Hospital Clinical Laboratory, Departments of Medicine and Pathology-Laboratory Medicine, Madison, WI 53706

Accuracy in clinical chemistry analyses remains vague and difficult to quantitate in individual samples. The analytical chemistry principles of standard addition, interference studies, reagent blank studies, the use of National Bureau of Standards’ Standard Reference Materials (NBS-SRMs) and the development of definitive analytical methodology have contributed significantly to the improvement of the accuracy of clinical chemistry methods in the last 25 years. However, absolute accuracy for all biological samples remains an unattainable goal for the Field of clinical chemistry analysis.

Clinical chemistry as a distinct field started to evolve principally from biochemistry shortly after the turn of this century. Analytical chemistry method development at that time was hindered by the unavailability of highly pure and stable chemical standards. Frequently, clinical correlation of laboratory numbers to the patient’s medical condition was one of the major pieces of information that was used to assess the accuracy of the laboratory measurement. As commercial sources of chemical standards became available, products from different companies were compared and exchange of samples between laboratories (“round robins”) revealed many accuracy and calibration problems with the analytical measurements. In the late 1950’s when the AutoAnalyzer became commercially available from the Technicon Corporation, common calibration materials and reagents from Technicon greatly reduced the bias in laboratory results between different hospital laboratories. In addition, the precision of the analytical methods dramatically improved as a result of the Technicon mechanization of the measurement process. However, this increase in precision did not bring with it the expected improvement in accuracy.

The focused emphasis on increased accuracy in clinical chemistry analysis in the 1960’s was initiated and led by the National Bureau of Standards (NBS). In 1966, Dr. W. Wayne Meinke presented a talk at the national meeting of the American Association for Clinical Chemistry (AACC) on “Standards.” In June of 1969, the National Institutes of Health (NIH) announced they would award 3 million dollars to NBS for the development of SRMs for the clinical chemistry laboratory. A progress report of the research at NBS was written by Dr. Meinke and appeared in *Analytical Chemistry* [1].

With the availability of the NBS-SRMs, clinical chemists quickly found they were unable to obtain the correct answers for the highly purified reference materials using their usual daily clinical laboratory instruments and methods. Further, if they used the NBS-SRM materials to calibrate the methods in daily use, the “normal ranges” would have to be significantly changed from the long established textbook values. This was not acceptable in most laboratories.

The Clinical Laboratory Improvement Acts of 1969 and 1973 and the Medical Device Law of 1976 forced accuracy issues and changes upon the field of clinical chemistry. This demand occurred at a time when the capabilities for improved accuracy were limited and when this goal was difficult to achieve.

The original intention of the improvement acts was to make sure that Medicare and Medicaid payments were being made for quality health care delivery. It appears that one of the achievements of the laws was the limitation of interstate commerce of samples and laboratory results. These laws also caused the proliferation of proficiency testing as a measure of the quality of a laboratory. We have since learned that proficiency testing is not a dependable way to detect inaccurate laboratory results.

The Medical Device law of 1976 produced a flurry of activity at the Food and Drug Administration (FDA) causing them to work on “reference methods” of analysis, while at the NBS, efforts were directed at development of “definitive methods.” The definitive methods have been, and continue to be, successfully used and continue to be developed. These definitive methods have improved the quality of data and information that proficiency testing programs can obtain. The development of reference methods has not produced similar successes.

In the 1980’s the FDA has had as one of its missions the classification and pre-market evaluation of many commercial products used in clinical laboratories. Initially, for analytical and instrumental methods of analysis, a requirement of FDA was that a new proposed method must produce results that have a high positive correlation with the previously accepted physical-chemical method of analysis. In the area of drug analysis, the accepted method was often gas or high pressure liquid chromatography, occasionally with mass spectrometry. After many months of testing, most of the original immunochemical methods presented to FDA did not meet the expected performance standards. The FDA developed a large backlog of 510(k)’s and the commercial companies discovered that by presenting their new methodology as “substantially equivalent” the approval rate increased and delay at FDA was shortened. This procedural change by the manufacturers and at FDA rapidly improved the efficiencies of handling the 510(k)’s at FDA.

Today, decentralization of clinical chemistry testing is occurring rapidly and estimates for the size of the business of home testing and doctor’s office testing is at $400 million per year. Establishment and maintenance of high accuracy in these decentralized facilities are increasingly difficult to achieve.

Nerve tissue aluminum, urine cadmium, whole blood cyclosporine, serum theophylline, and serum cholesterol are five examples of analytes where methodologie difficulties and/or lack of accuracy can produce analytical results of poor quality. In the graphite furnace atomic absorption spectrometric (GFAAS) method of measuring aluminum in neuronal tissue [2] variations in the matrix, even after digestion, apparently cause inaccuracy problems [3]. In the GFAAS method of measuring cadmium in urine, the spectral interference of sodium in the sample is the most difficult matrix problem that can contribute to inaccurate results [4]. In the HPLC method of measuring whole blood cyclosporine [5], many metabolites, interferences due to hemoglobin and bilirubin, and nonreproducibility of the sample preparation steps all contribute to inaccurate results. Using immunochemical methods to measure serum theophylline creates inaccurate laboratory data when the samples are from patients that have renal failure. In the case of serum cholesterol analysis, the lower-than-desired accuracy is principally due to matrix interferences in the routine methodology used in clinical laboratories.

The above examples of inaccuracies would be much more serious but for the ways that physicians use laboratory results. If the quality of the analytical results are high then much more effort is invested in attempting to make logical decisions with that data. If the quality of the laboratory is poor then the decision may be made to ignore the laboratory result and proceed to “care for the clinical condition of the patient.” Alternately, the physician may decide to reorder the test a second time to see if a statistically different result is obtained.

Most interferences in analytical methodologies can be eliminated by a chromatographic sample preparation step prior to analysis. However, chromatographic separations are not usually automated, are poorly mechanized, and add time and expense to the analysis. In a high volume service clinical chemistry laboratory, the extra step required by a chromatographic separation is very undesirable. Therefore, today many inaccuracies remain with the routine methods used rather than adapt a cure for the accuracy problem by adding a manual chromatographic sample preparation step.

In conclusion, the development of highly accurate instrumental methods of chemical analysis for the clinical laboratory remains a very difficult task. When one includes the nonanalytical priorities in method development, then decreasing emphasis on accuracy is often accepted by members of this field. Decentralization of the service laboratory causes some additional reassessment of the criteria for test methods. A list of desired characteristics for test methodology can be created. That list of performance criteria in decreasing order of importance is: 24-hour availability of results, minimum sample pretreatment, wide selection of different types of tests, low operator skill, high precision, low total cost per analysis, short throughput time, and high accuracy.

Analytical chemistry has much to contribute to the field of clinical chemistry analysis but ways must be found to produce highly accurate test results while lowering the cost and decreasing the difficulty of the analysis. We as analytical chemists have addressed the cost issue but have not contributed much to the improvement of accuracy while simplifying the technical difficulty of the clinical laboratory tests. This last task then remains as a challenge for the near future.
